# The enhancement of combination of berberine and metformin in inhibition of DNMT1 gene expression through interplay of SP1 and PDPK1

**DOI:** 10.1111/jcmm.13347

**Published:** 2017-08-25

**Authors:** Fang Zheng, JingJing Wu, Qing Tang, Qian Xiao, WanYin Wu, Swei Sunny Hann

**Affiliations:** ^1^ Laboratory of Tumor Biology Guangdong Provincial Hospital of Chinese Medicine The Second Clinical Medical Collage Guangzhou University of Chinese Medicine Guangzhou Guangdong Province China; ^2^ Department of Medical Oncology Guangdong Provincial Hospital of Chinese Medicine The Second Clinical Medical Collage Guangzhou University of Chinese Medicine Guangzhou Guangdong Province China

**Keywords:** Berberine, Metformin, NSCLC, PDPK1, SP1, DNMT1

## Abstract

Berberine (BBR), one of active alkaloid found in the rhizome, exhibited anti‐cancer properties. We have showed that BBR inhibited growth of non‐small cell lung cancer (NSCLC) cells through mitogen‐activated protein kinase (MAPK)‐mediated increase in forkhead box O3a (FOXO3a). However, the in‐depth mechanism underlying the anti‐tumor effects still remained to be elucidated. Herein, we further confirmed that BBR not only induced cell cycle arrest, but also reduced migration and invasion of NSCLC cells. Mechanistically, we observed that BBR reduced 3‐phosphoinositide‐dependent protein kinase‐1 (PDPK1) and transcription factor SP1 protein expressions. Exogenously expressed SP1 overcame BBR‐inhibited PDPK1 expression. Moreover, BBR inhibited DNA methyltransferase 1 (DNMT1) gene expression and overexpressed DNMT1 resisted BBR‐inhibited cell growth. Intriguingly, overexpressed PDPK1 antagonized BBR‐inhibited SP1 and DNMT1 expressions. Finally, metformin enhanced the effects of BBR both *in vitro* and *in vivo*. Collectively, we observe that BBR inhibits proliferation of NSCLC cells through inhibition of SP1 and PDPK1; this results in a reduction of DNMT1 expression. The interplay of PDPK1 and SP1 contributes to the inhibition of DNMT1 in response to BBR. In addition, there is a synergy of BBR and metformin. This study uncovers a new mechanism of BBR in combination with metformin for NSCLC‐associated therapy.

## Introduction

Lung cancer is the most common and lethal malignancy worldwide. Among this, the majority of patients are NSCLC and demonstrate with advanced stages 1. Despite substantial advances in diagnosis and management, the outcomes for this malignancy are still unfavourable. Studies have demonstrated potential benefits using other potential therapeutics in patients with lung cancer 2, 3. Chinese herbal medicine and its components play an important role in cancer patients in reducing other treatment related complications, and improving quality of life (QOL) including lung malignancy 4, 5. However, the mechanisms by which these herbal components in associating with the therapeutic efficiency in the treatment of lung cancer still remains to be elucidated.

Chemical compounds derived from plants with wide range of pharmacological and biochemical effects have been used over decades for a number of diseases including cancer. A large growing body of evidence have demonstrated that BBR, an active alkaloid from the rhizome and a number of Chinese herbs, has anticancer effects 6, 7. BBR has been reported to inhibit cell proliferation and induce apoptosis in multiple cancer types 8, 9, 10, 11. For example, BBR demonstrated the abilities in inhibition of migratory and invasion of prostate cancer cells through regulation of epithelial‐to‐mesenchymal transition (EMT)‐related genes, and this may provide potential therapeutic targets in patients with prostate cancer 12. Moreover, BBR reduced breast cancer cell growth by generating reactive oxygen species *via* mitochondrial‐related apoptotic pathway 13. Moreover, BBR blocked doxorubicin (DOX)‐induced activation of signal transducer and activator of transcription 3 (STAT3) and enhanced the cytotoxic effect of DOX in human lung cancer cells 14. We previously showed that BBR inhibited growth and induced apoptosis of NSCLC cells through p38α MAPK‐mediated increase in p53 and forkhead O transcription factor 3 alpha (FOXO3a) proteins 15. Melatonin, a known natural antioxidant, showed to enhance the anti‐lung cancer effects of BBR through activating caspase and inhibiting transcription factors activator protein‐2 beta (AP‐2β), nuclear transcription factor κB (NF‐κB)/ cyclooxygenase 2 (COX‐2) and Akt/ extracellular signal‐regulated kinase (ERK) signalling pathways 8. Thus, combination of conventional therapy with other potential herbal agents may improve the treatment outcome for lung cancer patients. Nevertheless, the detailed molecular mechanism underlying this potential synergy still required to be elucidated.

Phosphoinositide‐dependent kinase 1 (PDPK1) is a proximal signalling molecule of phosphatidylinositol 3‐kinase (PI3‐K) of AGC kinase family. As a key regulator of metabolism and metastatic potential in many cancer types, PDPK1 activates multiple downstream effectors and implicates in diverse biological functions 16, 17. The pleiotropic capacity of PDPK1 makes it a promising molecular and therapeutic target for various types of cancer including lung 18, 19, 20, 21. Study observed that miRNAs such as miR‐138 could bind to the 3′‐UTR of PDPK1, thereby reducing expression of PDPK1. And knockdown of PDPK1 significantly repressed the growth of lung cancer cells, suggesting that miR‐138 inhibited cell proliferation by targeting PDPK1 in lung cancer cells. Thus, miR‐138 and PDPK1 might predict the prognosis and both together represented promising biomarkers in progression and survival in patients with lung cancer 20. We previously demonstrated that a peroxisome proliferator‐activated receptor gamma (PPARγ) ligand ciglitazone inhibited PDPK1 expression through AMP‐activated protein kinase alpha (AMPKα)‐induced increase in transcription factor Egr‐1 expression and bind to the PDPK1 gene promoter. Activation of AMPKα by metformin (MET) enhanced the effect of ciglitazone on inhibition of NSCLC cell growth 22. Despite these findings, the true role and function of PDPK1 in the tumorigenesis, growth and progression of lung cancer still required to be determined.

The process of DNA hypermethylation, which is initiated by DNA methyltransferases (DNMTs), is considered as one of the main reasons for inhibition of tumour‐suppressing genes 23, 24. Three active mammalian DNMTs, such as DNMT1, DNMT3a and DNMT3b, have been identified. Among these, DNMT1 is the most common one in humans 25. DNMT1 has been shown to be involved in the various biological functions including tumour growth, progression and survival 26, 27, 28. Several lines of evidence have demonstrated that increased expression of DNMT1 is existed in different cancers including lung and that targeting DNMT1 suppresses cancer cell growth 29, 30. The interaction between menin, the product of the Men1 gene and DNMT1 reversibly influenced growth of pancreatic cancer cells through affecting downstream of Hedgehog pathways, implying that the complex of Hedgehog/DNMT1/menin axis is potential molecular targets for the treatment of pancreatic cancer 27. Study showed that interleukin 6 (IL‐6) increased the expression of DNMT1 in lung cancer cells, and that knockdown of DNMT1 reversed IL‐6‐mediated hypermethylation of cell cycle regulatory genes and enhancement of lung cancer stem‐like properties, implying that IL‐6‐mediated pathway increased DNMT1 expression and enhanced lung cancer stem cell (CSC) proliferation 26. One recent study demonstrated a link of BBR and DNMT1, and showed that miR‐152 was regulated by DNMT1 and that BBR could influence the expression of DNMT1 and other genes in colon tissues from neonatal rats, thereby regulating miR‐152 expression. This might provide some evidence for the potential for colon cancer treatment 31. Report suggested that the reciprocal targeting of protein kinases and DNMT1 may be considered as a novel strategy for substantial therapeutic responses in lung cancer as well 32. Thus, inhibition of DNMT1 could be a promising therapeutic potential for lung cancer. At present, the links between the PDPK1 and DNMT1 have not been well studied.

In this study, we examined potential mechanism by which BBR alone and combining with MET inhibited lung cancer cell proliferation. Our results demonstrate that BBR inhibits growth, migration and invasion of NSCLC cells through inhibition of SP1 and PDPK1; subsequently, this results in the inhibition of DNMT1 gene expression. In addition, there is a potential synergy of BBR combining with MET in this process.

## Materials and Methods

### Reagents

Monoclonal antibodies against DNMT1 and SP1 were purchased from Cell Signaling Technology Inc. (Beverly, MA, USA). The PDPK1, GAPDH, total Akt and the phosphor‐form (Ser473) antibodies were obtained from Abcam (Cambridge, MA, USA). MET was purchased from Enzo (Grand Island, NY, USA), MTT powder was obtained from Sigma‐Aldrich (St. Louis, MO, USA), Cell Proliferation ELISA and BrdU (colorimetric) Kits were obtained from Roche (Basel, Switzerland). Control (pCMV6‐AC or pCMV6‐GFP), DNMT1, PDPK1 and Akt overexpression plasmids and PDPK1 promoter constructs were purchased from OriGene Technologies (Rockville, MD, USA). BBR was purchased from Chengdu Must Bio‐technology Company (Chengdu, Sichuan, China). Agents were dissolved with DMSO, and freshly diluted to the working concentration with medium before use.

### Cell lines and cultures

Human NSCLC cells (A549 and H1975) were obtained from the Chinese Academy of Sciences Cell Bank of Type Culture Collection (Shanghai, China). The cells were cultured at 37°C in a humidified atmosphere containing 5% CO2. The culture medium consisted of RPMI 1640 medium obtained from Life Technologies (GIBCO) supplemented with 10% (v/v) heat‐inactivated foetal bovine serum (GIBCO, Grand Island, NY, USA), 100 μg/ml streptomycin and 100 U/ml penicillin.

### Cell viability assay

We used 3‐(4, 5‐dimethylthiazol‐2‐yl)‐2, 5‐diphenyltetrazolium bromide (MTT) method to determine cell viability as described previously 33. NSCLC cells (6 × 10^3^ cells/96‐microwell plate) were treated with increasing concentrations of BBR for up to 72 hrs. Afterwards, 10 μl MTT solution (5 g/l) was added and incubated at 37°C for an additional 4 hrs. Finally, after removing the supernatant, 150 μl solvent dimethyl sulphoxide (DMSO) was added and oscillated for 10 min. Afterwards, absorbance at 570 nm was determined through the use of ELISA reader (BioTek, Epoch, VT, USA). Cell viability (%) was calculated as follows: (absorbance of test sample/absorbance of control) ×100%.

### Cell proliferation assay

NSCLC cells were cultivated in 96‐micro‐well plates at a density of 6 × 10^3^ cells/well and treated with increasing concentrations of BBR for 48 hrs. For the last 16 hrs, cells were added with BrdU labelling solution (final concentration: 10 μM BrdU). Subsequently, the incorporation rate of BrdU was determined using a commercial enzyme‐linked immunosorbent assay (ELISA) kit (Roche, Indianapolis, IN USA). A colorimetric reaction with tetramethylbenzidine (TMB) as a substrate gave rise to a reaction product measured at 370 nm using a scanning multi‐well spectrophotometer (BioTek).

### Cell cycle analysis

NSCLC cells (3 × 10^5^ cells/well) were cultured in 6‐well plates and treated with increased doses of BBR for 24 hrs. Then, the cells were harvested and resuspended in 70% pre‐cold ethanol for 24 hrs at −20°C, followed by incubating with 0.1% sodium citrate containing propidium iodide (PI) 0.05 mg and 50 μg RNase for 30 min. Finally, the cell cycle analysis was detected by flow cytometry (FC500; Beckman Coulter, Brea, CA USA), and the proportion of cells within the G0/G1, S and G2/M phases of the cell cycle were analysed using the Multi‐Cycle AV DNA Analysis software (Phoenix Flow Systems, Inc., San Diego, CA, USA).

### Wound‐healing assay

Wound‐healing assay was used to examine NSCLC cells migration ability. The cells were grown to 100% confluence in six‐well plates and incubated with starvation medium overnight. Cell monolayers were wounded with a sterile 200 μl pipette tip and washed to remove detached cells. Cells were treated with indicated doses of BBR for 24 hrs; afterwards, the medium was replaced with PBS, and the wound gap was observed and photographed using an Olympus microscope fitted with digital camera (Olympus, Tokyo, Japan).

### Cell invasion assays

The invasion assays were processed by using of the Transwell plate (Corning, Corelle NY, USA) with 10 mm diameter and 8 μM pore size polycarbonate membrane. Before the experiment, the Matrigel (BD, Franklin Lakes, NJ, USA) was diluted to eightfold, which was injected into the upper chamber of transwell system. The lower chamber was added into 500 μl of cell culture medium with 20% FBS. Afterwards, cells were diluted to 0.5 × 10^6^/ml and pre‐treated with BBR (50 μM), and add 200 μl A549 cells suspension into the Matrige. Following 24‐hrs incubation, cells remained in upper membrane were wiped, while cells migrated or invaded were fixed with 4% paraformaldehyde, stained with 0.1% crystal violet, and counted under a microscope (Olympus Corporation).

### Quantitative real‐time PCR (qRT‐PCR)

A quantitative real‐time PCR assay was developed for the detection of the expression of DNMT1. GAPDH was used as an endogenous control. The primers used in this study were designed as follows: DNMT1: forward 5′‐GTGAGGACATGCAGCTTTCA ‐3′ and reverse 5′‐TGCTGCCT TTGATGTAGTCG‐3′; GAPDH (used as an internal control): forward 5′‐CTCCTCCTGTTCGACAGTCAGC‐3′ and reverse 5′‐CCCAATACGACCAAATCCGTT‐3′. Total RNA was extracted using the TRIzol solution, and the first‐strand cDNA was synthesized from total RNA (1 μg) by reverse transcription using PrimeScript™RT Reagent Kit (Takara Bio Inc., Kyoto Japan) according to the manufacturer's instructions. Quantitative real‐time PCR was performed in a 20 μl mixture containing 2 μl of the cDNA preparation using SYBRPremix Ex Taq™II (Tli RNaseH Plus) Kit (Takara Bio Inc.) on an ABI 7500 Real‐Time PCR System (Applied Biosystems, Grand Island, NY, USA). The PCR conditions were as follows: 30 sec. at 95°C, followed by 40 cycles of 5 sec. at 95°C and 34 sec. at 60°C. Each sample was tested in triplicate. Relative expression of DNMT1 was calculated as ΔC_t_, measured by subtracting the C_t_ of the GAPDH.

### Western blot analysis

The detailed method was based on previous report 33. Whole cell lysates containing same amount of protein were solubilized in 3× SDS‐sample buffer and separated on 10% SDS polyacrylamide gels. PVDF Membranes (Millipore, Billerica, MA, USA) were incubated with antibodies against DNMT1, SP1, PDPK1 and GAPDH (1:1000). The membranes were washed and incubated with a secondary goat antibody raised against rabbit IgG conjugated to horseradish peroxidase (Cell Signaling). The membranes were washed again and transferred to freshly made ECL solution (Millipore) and observed, recorded the signals using the Gel Imagine System (Bio‐Rad, Hercules, CA, USA).

### Transient transfection assays

The detailed procedure was reported previously 33. NSCLC cells (5 × 10^5^ cells/well) were seeded in 6‐well dishes and grown to 70–80% confluence. For each well, 2.5 μg pcDNA 3.1 or SP1 plasmid DNA, kindly provided by Dr. Thomas E. Eling (NIEHS, Research Triangle Park, NC, USA) 34, and 1.5 μg pCMV6‐AC/GFP or DNMT1, Akt expression vectors or 2.5 μg PDPK1 plasmid were transfected into the cells using the lipofectamine 3000 reagent according to the manufacturer's instructions for up to 24 hrs, followed by treating with BBR for an additional 24 hrs. In separated experiment, cell were transfected with pEZX‐PG04‐DNMT1 promoter construct linked Gaussia luciferase (GLuc) gene and secreted alkaline phosphatase (SEAP) internal control obtained from GeneCopoeia, Inc. Secrete‐Pair Dual Luminescence Assay Kit (GeneCopoeia, Inc Rockville, MD, USA) was used to determine luciferase activities, which was normalized with SEAP within each sample.

### Tumour xenografts and bioluminescent imaging

Animal study was approved and carried out in accordance with the recommendations in the Guide for the Care and Use of Laboratory Animals in cancer research of Use Committee of Guangdong Provincial Hospital of Chinese Medicine. A total of 24 eight‐week‐old female nude mice obtained from Guangdong Provincial Research Center for Laboratory Animal Medicine (Foshan, Guangdong, China) were maintained at the Animal Center of Guangdong Provincial Hospital of Chinese Medicine. A549 cells carrying luciferase report gene (A549‐Luc, obtained from the Guangzhou Land Technology Co., Guangzhou, China) in 0.2 ml medium (phenol red free RIPM 1640 with 2% FBS) were injected subcutaneously into the nude mice. Xenografts were allowed to grow for over 1 week when the tumour diameters reached 3 mm × 4 mm made with callipers. Mice were randomly divided into following groups: vehicle (DMSO), BBR (10 mg/kg, ip, QOD)^8^, MET, (250 mg/kg, ig, QD) 35 and the combination groups for up to 3 weeks. For bioluminescence imaging (BLI) procedure, the mice were anaesthetized by inhalation of 2% isoflurane at the end of experiment. Each set of mice were injected with 150 mg/kg D‐luciferin (Caliper Life Sciences, Hopkinton, MA, USA) in approximately 200 μl. Imaging and quantification of signals (photons/sec.) were obtained using the IVIS‐200 Imaging System (Xenogen Corporation, Berkeley, CA, USA). Tumour volume measurements were calculated using the formula for an oblong sphere: volume = (width2 × length). The bodyweights of mice were measured twice a week. All mice were killed on day 21 by cervical dislocation with minimum suffering in accordance with the Guide for the Care and Use of Laboratory Animals. The corresponding extracted xenografts were processed for detecting the DNMT1, SP1, PDPK1 proteins by Western blot.

### Statistical analysis

The majority of experiments were repeated a minimum of three times. All data are expressed as mean ± S.D. Differences between groups were assessed by one‐way or two‐way anova, and significance of difference between particular treatment groups was analysed using Dunnett's multiple comparison tests by GraphPad Prism software version 5.0. (Graph Pad software, Inc. San Diego, CA, USA, www.graphpad.com). Asterisks showed in the figures indicate significant differences of experimental groups in comparison with the corresponding control condition. *P*‐values <0.05 were considered statistically significant.

## Results

### BBR inhibited growth, migration, invasion, and induced cell cycle arrest in lung cancer cells

**Figure 1:**
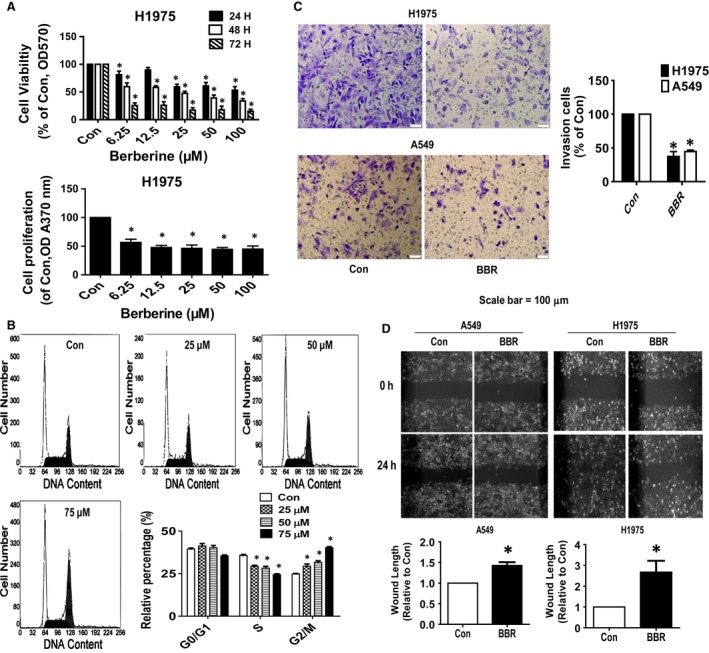
BBR inhibited growth, migration, invasion, and induced cell cycle arrest in lung cancer cells. (**A–B**) H1975 cells were treated with increased concentrations of BBR for up to 72 hrs to examine the cell viability by MTT and colorimetric BrdU ELISA methods as described in the Materials and Methods section. (**C**) H1975 cells were treated with BBR (50 μM) for up to 24 hrs. Shown are representative images of fixed and crystal violet‐stained H1650 cell invasion on the Matrigel‐coated inserts in the presence of vehicle control (Con), and 50 μM BBR. Scale bar = 100 μm. *indicates a significant difference from the control group (*P* < 0.05). (**D**) Cell migration ability was determined by scratching confluent A549 and H1975 cell monolayers with a pipette tip. Cells were incubated with BBR (50 μM) for 24 hrs. The extent of migration in each sample was photographed by Axiovert 200 microscope (magnification, ×100) and expressed as relative to control (0 hr). Values are given as the mean ± S.D. from three independent experiments. *indicates a significant difference from the control group (*P* < 0.05).

We previously showed that BBR inhibited growth of human lung cancer A549 cells 15. In the current study, we further substantiated the effect of inhibition affected by BBR. As expected, we observed that BBR significantly inhibited growth of an additional NSCLC cell H1975 (Fig. 1A) using both MTT and colorimetric BrdU ELISA methods. Next, the experiments of cell cycle phase distribution treated with increased doses of BBR for 24 hrs showed that BBR significantly increased the proportion of cells at G2/M phase and reduced the proportion of cells at S phases (Fig. 1B), suggesting that BBR induced cell cycle arrested in G2/M phase in H1975 cells. Moreover, we studied *in vitro* invasive properties of lung cancer cells in responding to BBR using matrigel‐coated transwell experiments. The results indicated that BBR inhibited invasion of H1975 and A549 cells (Fig. 1C). Furthermore, BBR significantly reduced wound gap closure compared to the untreated control cells as determined by wound healing assays, indicating reduction of migration by BBR in H1975 and A549 cells (Fig. 1D). The results above confirmed the multiple anti‐lung cancer effects of BBR in this process.

### BBR decreased protein expression of PDPK1 and SP1

**Figure 2:**
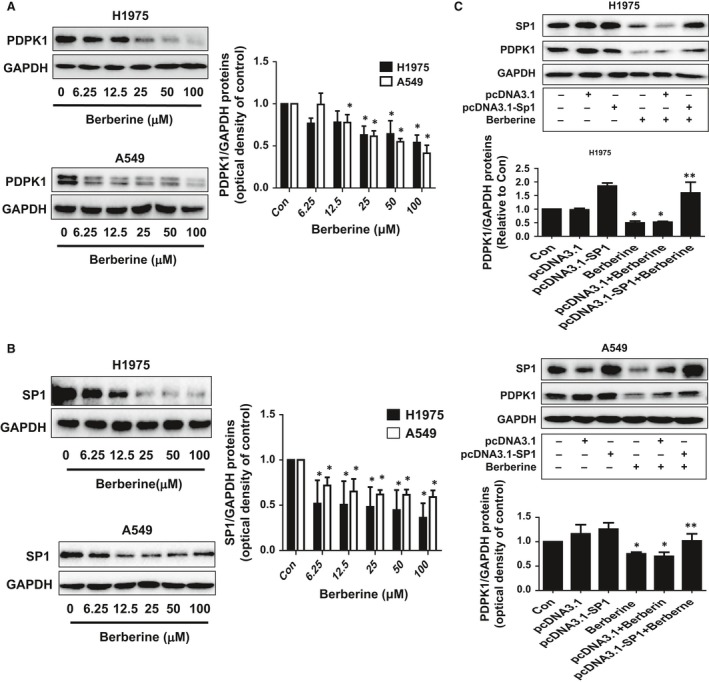
BBR decreased protein expression of PDPK1 and SP1. (**A–B**) A549 and H1975 cells were exposed to increased concentrations of BBR for 24 hrs, followed by measuring PDPK1 and SP1 proteins by Western blot. (**C**) A549 and H1975 cells were transfected with the control or expression constructs of SP1 for 24 hrs before exposing the cells to BBR for an additional 24 hrs. Afterwards, SP1 and PDPK1 protein expressions were determined using Western blot. Figures are representative cropped gels/blots that have been run under the same experimental conditions. Values are given as the mean ± S.D. from three independent experiments. *indicates a significant difference from the control group (*P* < 0.05).

We next explored the molecular mechanism by which BBR inhibited cell growth. First, in order to identify the relevant targets of BBR in this process, we began to investigate the role of transcription factor SP1 and one key kinase PDPK1, both of which have been involved in the tumour growth and progression 16, 17, 36. Our results showed that BBR reduced protein expressions of SP1 and PDPK1 in a dose‐dependent manner in A549 and H1975 cells (Fig. 2A and B). Next, we further examined the potential interaction between the SP1 and PDPK1 signalling. As shown in Figure 2C, exogenously expressed SP1 reversed the BBR‐reduced PDPK1 protein expression. This result indicated that SP1 regulated PDPK1 expression in this process.

### BBR inhibited DNMT1 mRNA, protein and promoter activity

**Figure 3:**
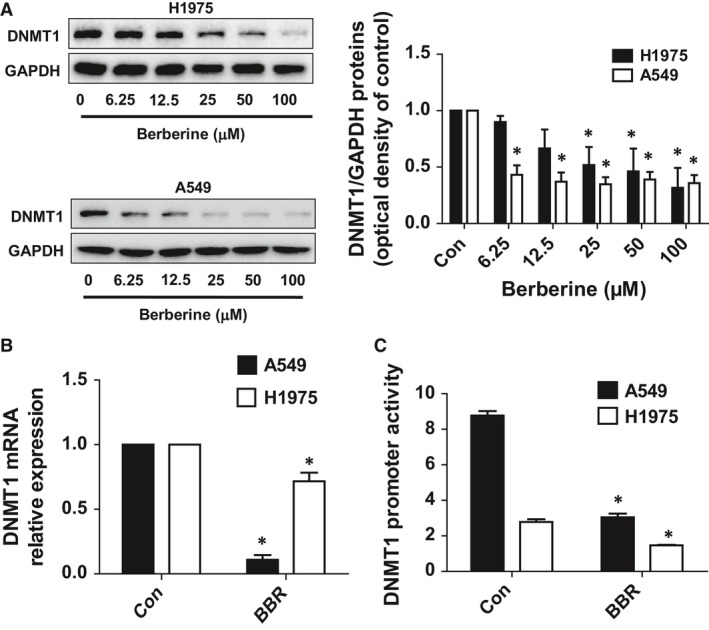
BBR inhibited DNMT1 mRNA, protein and promoter activity. (**A‐B**) A549 and H1975 cells were exposed to increased doses of BBR for 24 hrs, followed by measuring the protein, mRNA expressions of DNMT1 by Western Blot and qRT‐PCR, respectively. Figures are representative cropped gels/blots that have been run under the same experimental conditions. (**C**) A549 and H1975 cells were transfected with wild‐type human DNMT1 promoter reporter construct ligated to luciferase reporter gene and internal control secreted alkaline phosphatase for 24 hrs, followed by treating with BBR for an additional 24 hrs. Afterwards, the promoter activities were determined using the Secrete‐Pair Dual Luminescence Assay Kit as described in the Materials and Methods section. Values in bar graphs were given as the mean ± S.D. from three independent experiments performed in triplicate. * indicates significant difference as compared to the untreated control group (*P* < 0.05).

To dissect the mechanism and gain insight into the biological significance of the interaction between SP1 and PDPK1, we further elucidated the potential downstream effectors. In this study, we have deciphered the role of DNMT1, which is the target of SP1 37 and recently have been reported to be associated with PDPK1 signalling 38. We showed that BBR inhibited the protein, mRNA and promoter activity of DNMT1 in H1975 and A549 cells as determined by Western Blot, qTR‐PCR and Secrete‐Pair Dual Luminescence Assay Kit, respectively (Fig. 3A–C).

### Overexpression of DNMT1 resisted cell growth inhibition affected by BBR

**Figure 4:**
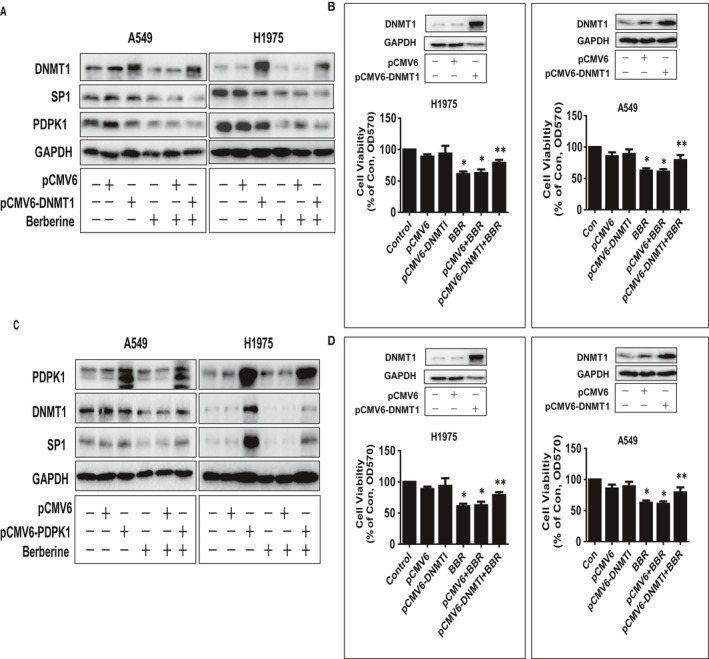
Overexpression of DNMT1 resisted cell growth inhibition affected by BBR. (**A**) A549 and H1975 cells were transfected with the control (pCMV6) or expression constructs of DNMT1 for 24 hrs before exposing the cells to BBR (50 μM) for an additional 24 hrs. Afterwards, PDPK1, SP1 and DNMT1 proteins were determined by Western blot. Figures are representative cropped gels/blots that have been run under the same experimental conditions. (**B**) A549 and H1975 cells were transfected with the control or expression constructs of DNMT1 for 24 hrs before exposing the cells to BBR (50 μM) for an additional 48 hrs. Afterwards, the cell viability was determined using the MTT assay as described in the Materials and Methods section. Insert on the upper panel represented the protein levels of DNMT1 as determined using Western blot. GAPDH was used as internal control. (**C**) A549 and H1975 cells were transfected with the control (pCMV6) or expression constructs of PDPK1 for 24 hrs before exposing the cells to BBR (50 μM) for an additional 24 hrs. Afterwards, PDPK1, SP1 and DNMT1 proteins were determined by Western blot. GAPDH was used as control. Figures are representative cropped gels/blots that have been run under the same experimental conditions. (**D**) A549 and H1975 cells were transfected with the control (pCMV6) or expression constructs of PDPK1, and wild‐type human DNMT1 promoter reporter construct ligated to luciferase reporter gene and internal control secreted alkaline phosphatase for 24 hrs, followed by treating with BBR for an additional 24 hrs. Afterwards, the promoter activities were determined using the Secrete‐Pair Dual Luminescence Assay Kit as described in the Materials and Methods section. Insert on the upper panel represented the protein levels of PDPK1 as determined using Western blot. Values in bar graphs were given as the mean ± S.D. from three independent experiments. * indicates significant difference as compared to the untreated control group (*P* < 0.05). ** indicates significant difference from the BBR treated alone (*P* < 0.05).

In order to further interrogate the importance of DNMT1 expression and the ability of DNMT1 to influence the expression of SP1, PDPK1, and regulate cell growth, the exogenously expressed DNMT1 gene were transfected into the cells. We observed that while overexpressed DNMT1 had little effect on influencing the BBR‐reduced SP1 and PDPK1 protein levels, it significantly overcame BBR‐inhibited growth of NSCLC cells (Fig. 4A and B). On the contrary, exogenously expressed PDPK1 appeared to resist BBR‐inhibited DNMT1 and SP1 protein expressions (Fig. 4C). It also antagonized BBR‐reduced DNMT1 promoter activity (Fig. 4D). The findings above suggested that the mutual regulation of SP1 and PDPK1 could reduce DNMT1 expression and that inhibition of DNMT1 at translational and transcriptional levels by BBR was involved in the inhibition of NSCLC cell growth.

### The synergistic effects of BBR and MET

**Figure 5:**
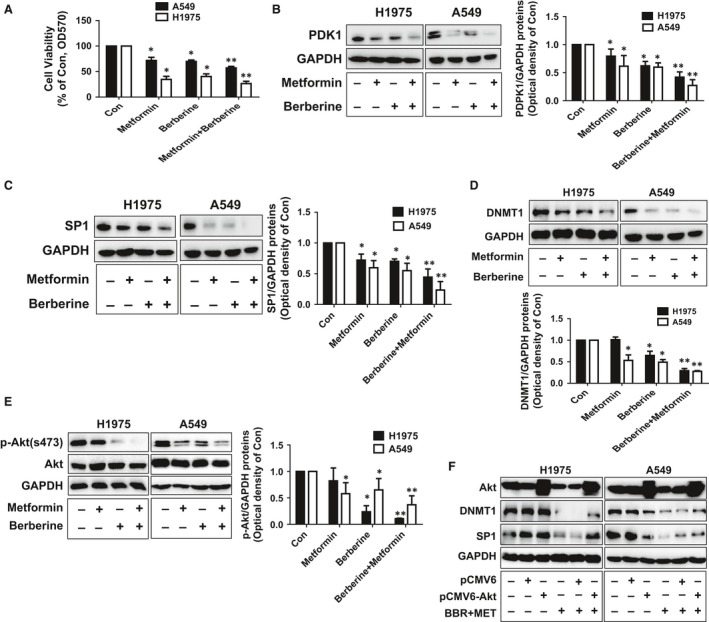
The synergistic effects of BBR and MET. (**A**) A549 and H1975 cells were exposed to BBR (50 μM) and MET (10 mM) for 24 hrs. Afterwards, the cell viability was determined using the MTT assay as described in the Materials and Methods section, and was expressed as percentage of control in the mean ± S.D. of three separate experiments. (**B–D**) A549 and H1975 cells were exposed to BBR (50 μM) and MET (10 mM) for 24 hrs, followed by measuring the protein expression of SP1, PDPK1 and DNMT1 by Western blot. (**E**) A549 and H1975 cells were exposed to BBR (50 μM) and MET (10 mM) for 24 hrs, followed by measuring the phosphorylation and protein expression of Akt by Western blot. The figures are representative cropped gels/blots that have been run under the same experimental conditions. (**F**) A549 and H1975 cells were transfected with the control (pCMV6) or expression constructs of Akt for 24 hrs before exposing the cells to BBR (50 μM) and MET (10 mM) for an additional 24 hrs. Afterwards, Akt, SP1 and DNMT1 proteins were determined by Western blot. GAPDH was used as control. Figures are representative cropped gels/blots that have been run under the same experimental conditions. * indicates significant difference as compared to the untreated control group (*P* < 0.05); ** indicates significance of combination treatment as compared to BBR alone (*P* < 0.05). The bar graphs represent the mean ± S.D. of PDPK1/SP1/DNMT1/GAPDH of three independent experiments.

Metformin, a known AMPK activator and drug for the treatment of type 2 diabetes, has been shown to have anti‐tumour effects 39. Herein, we also asked whether combination of MET and BBR could have synergistic effects. We found that combing with MET showed to further enhance the effects of BBR on not only cell growth (Fig. 5A), but also the protein levels of SP1, PDPK1 and DNMT1 (Fig. 5B–D). This implied a potential synergy in this process. Because of the crucial role of PDPK1 downstream PI3‐K/Akt pathway in cancer growth and progression 21, 40 and the reported links of the Akt in regulation of SP1 and DNMT1 expressions in several cancer types in other studies 33, 38, 41, we then assessed the role of Akt signalling in this process. Our results showed that BBR and MET alone reduced the phosphorylation of Akt, and that more robust inhibition was observed in combination with MET and BBR treatment group in A549 and H1975 cells (Fig. 5E). Note that the total Akt had no changes by these treatments. Moreover, exogenously expressed Akt into the cells resisted the effects of combination of MET and BBR on reduction of SP1 and DNMT1 protein expressions in A549 and H1975 cells (Fig. 5F). Together, these findings above indicated that combination of MET and BBR reduced phosphorylation of Akt, and that inactivation of Akt involved in the combination of MET and BBR‐reduced expressions of SP1 and DNMT1 in this process.

### *In vivo* anti‐tumour efficacy of BBR in mice

**Figure 6:**
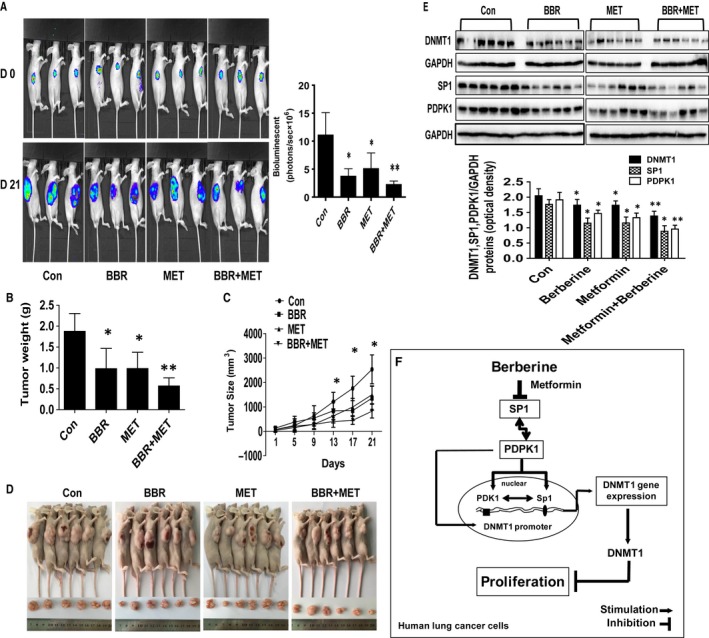
*In vivo* anti‐tumour efficacy of BBR in mice. Mice (*n* = 6/group) were divided into four groups [Con (saline), BBR alone (10 mg/kg), metformin (MET, 250 mg/kg) and the combination of BBR and metformin, which were given at the 1 week after tumour cells injection by either intraperitoneal injection or gavages for up to 21 days. (**A**) The xenografts were assessed by *in vivo* bioluminescence imaging at the day 0 and the end of the experiments (on day 21). The tumour growth was monitored by injecting luciferin in the mice followed by measuring bioluminescence and quantification of signals were controlled by the acquisition and analysis software as described in the Materials and Methods section. Representative images in all groups are shown. (**B‐C**) The xenografts were harvested on day 21, and the volume and weight of tumours were measured. (**D**) The photographs of vehicle‐ and drugs‐treated xenografts derived from nude mice are shown. (**E**) At the end of the experiments, xenografted tumours from the high dose and control groups were isolated from individual animals, and the corresponding lysates were processed for detecting PDPK1, SP1 and DNMT1 by Western blot. GAPDH was used as loading control. Figures are representative cropped gels/blots that have been run under the same experimental conditions. The bar graphs represented the tumour weight and volume of mice results of as mean ± S.D. * indicates the significant difference from the untreated control (*P* < 0.05). (**F**) The diagram shows that BBR inhibits growth of NSCLC cells through inhibition of SP1 and PDPK1; subsequently, this results in the reduction of DNMT1 gene expression. The interplay of PDPK1 and SP1 contribute to the inhibition of DNMT1 in response to BBR. In addition, there is a potential synergy of BBR and MET.

To further clarify the findings *in vitro,* we also assessed the effect of BBR on lung cancer growth *in vivo*. Mice bearing xenografted tumour was treated every other day for the control, BBR alone (10 mg/kg) 8 *via* intraperitoneal injection, MET, (250 mg/kg) by gavages 35 and the combination of BBR and MET 42 for up to 21 days. We found that the BBR, MET alone, and the combination of BBR and MET‐treated mice groups showed significant delayed tumour growth without any severe adverse responses as compared to that in the control group assessed by the Xenogen IVIS200 System (Fig. 6A). The difference in the levels of luciferase expression correlated with the tumour area. In addition, we observed significant reduction of the tumour weight and volume in the BBR, MET alone as compared to that in the control group. The combination group showed a greater suppression (Fig. 6B–D). By Western blot, fresh tumours harvested from the aforementioned experiment showed that BBR efficiently decreased SP1, PDPK1 and DNMT1 protein expressions *in vivo* as compared to that in the control one and that the combination of BBR and MET had even greater effects (Fig. 6E).

## Discussion

In this study, we investigated the consequence of human lung cancer cells to the treatment of BBR in the presence or absence of MET, confirming the anti‐lung cancer property of BBR in this process. Our current study showed that BBR inhibited growth of NSCLC cells through inhibition of SP1 and PDPK1; subsequently, this resulted in the reduction of DNMT1 gene expression. In addition, there is a potential synergy of BBR and MET. To our knowledge, this is for the first time to show that MET sensitized human lung cancer cell growth inhibition in response to BBR or *vice versa*.

We further explored the molecular mechanism underlying the anti‐lung cancer efficacy of BBR, studies observed signalling pathways and potential targets that were involved in the anti‐cancer responses of BBR in different cancer types 8, 43. We previously showed that BBR inhibited proliferation and induced apoptosis of NSCLC cells through p38α MAPK‐mediated increase in p53 and FOXO3a expressions 15. Herein, the current study indicated that reduction in transcription factor SP1 and one key member of AGC kinase family, PDPK1, was also involved in the inhibitory effect of BBR on NSCLC cell growth. As universal transcription factor, SP1 has been well considered to be involved in the growth, progression and metastasis of several cancer types 44. Moreover, the regulation of SP1 by BBR was also found in other studies; death‐domain‐associated protein (DAXX) regulated many cellular signalling pathways for cell growth and apoptosis, and through disrupting the association of SP1 binding to the DAXX gene promoter, BBR inhibited the DAXX promoter activity, thereby inducing human neuroblastoma cell apoptosis^45^. Another report observed that BBR suppressed hepatocellular carcinoma cell growth by upregulating miRNA‐22‐3p and downregulating the expression of SP1, thereby reducing the expressions of its downstream targets, CCND1, (cyclin D1), a oncogenic cell‐cycle‐related protein and BCL2, an apoptosis‐related protein 46. Hwangryunhaedok‐tang (HRT), one of a traditional medicine, which contained BBR, showed to inhibit the adipocyte differentiation by reducing adipocyte‐specific transcription factors, including PPARγ, CCAAT/enhancer binding protein beta (C/EBPβ), in 3T3‐L1 pre‐adipocytes through downregulation of multiple pathways including extracellular MAPK 1 (MEK1)/ERK1/2 and PDPK1/Akt 47. Intriguingly, we observed the novel links of SP1 and PDPK1 in the current study and suggested that there appeared a mutual regulation between SP1 and PDPK1, which resulted in the inhibition of DNMT1 expression and NSCLC growth affected by BBR. This novel interaction between SP1 and PDPK1 has not been shown so far. Thus, the mechanism underlying this interaction and subsequent outcomes required to be further determined. Nevertheless, our results have demonstrated important roles of these two tumour promoters in the growth and progression of lung cancer.

Furthermore, our observation has suggested a critical role of DNMT1 in mediating the effect of BBR on inhibiting growth of NSCLC cells. DNMT1 not only is a key maintenance methyltransferase 25 but also involves in tumour growth and progression 23, 26, 27, 32, 48. Several lines of evidence have demonstrated that increased expression of DNMT1 exists in several cancer types including lung and that targeting DNMT1 suppresses cancer cell growth 27, 29, 49, 50. Our results suggested that DNMT1 functioned as one of downstream effectors of SP1 and PDPK1, and that reduction in DNMT1 was involved in the BBR‐inhibited NSCLC cell growth. Studies have unveiled an important role for DNMT1 in modulating other gene expression through interacting with some transcription factors in other study 51. The modulation of SP1 and PDPK1 expression influenced the DNMT1 expression and functions have been reported in other studies as well. The SP family has been shown to bind to common cis‐elements in the promoter regions of various genes including DNMT1, thereby regulating DNMT1 expression 37, 52. SP1 modulated the expression of DNMT1 gene through mechanisms such as protein abundance, post‐translational modifications or interaction with other transcription factors in human acute myeloid leukaemia cells 52. One study demonstrated that p53, by forming a complex with SP1 on the DNMT1 gene promoter, reversely controlled DNMT1 expression; thus, deregulation of DNMT1 was associated with gain of transcriptional activation of SP1. This resulted in epigenetic alteration of multiple tumour suppressor genes and ultimately led to lung tumorgenesis 53. We previously showed that β‐elemene, a compound derived from Rhizoma zedoariae, inhibited NSCLC cell growth *via* extracellular signal‐regulated kinase1/2 (ERK1/2)‐ and AMPKα‐mediated inhibition of SP1, and this resulted in reduction in DNMT1 protein 33. In studying the potential mechanisms underlying the hypermethylation of tumour suppressor genes in helicobacter pylori (HP)‐induced gastric carcinoma, one study observed that interaction of PDPK1 and Akt caused to increase in Akt phosphorylation, thereby activating NF‐κB bound to DNMT1 promoter and increasing its expression in gastric carcinoma cells 38. However, less information showed the direct connection of PDPK1 and DNMT1, we believed that this is a first report, which provided the novel insight into the connection between PDPK1 and DNMT1 affected by BBR, and also highlighted the tumour promoter function of PDPK1 and DNMT1 that were involved in the anti‐tumour effect of BBR in this process. Nevertheless, the in‐depth mechanism of this interplay in mediating the anti‐tumour effect of BBR required to be further elucidated.

Moreover, our results unveiled at least an additive, or perhaps a synergistic effect of combination of BBR and MET in the inhibition of SP1, PDPK1 and DNMT1 expressions, and lung cancer cell growth, implying the potential new role and molecular mechanism in combination with BBR and MET in controlling NSCLC cell growth. MET, as a potential anticancer agent, has been shown to inhibit growth and induce apoptosis through AMPK‐dependent and AMPK‐independent signalling pathways in NSCLC cells 35, 54, 55. Substantial efficacy of MET and BBR in combining has been reported in other studies as well 56. One study found that BBR and MET inhibited mitogenic signalling in pancreatic ductal adenocarcinoma cells through AMPK‐dependent and AMPK‐independent pathways 42. In the current study, we reasoned that MET sensitized the effects of BBR on controlling NSCLC cell growth through enhancing the inhibition of PDPK1, SP1 and DNMT1 expressions, or *vice versa*. However, whether the effect of MET was depending on AMPK‐mediated pathway required to be determined. Moreover, because of the crucial role of PDPK1 downstream PI3‐K/Akt pathway in cancer growth and progression 21, 40 and the reported links of this pathway in the regulation of SP1 and DNMT1 expressions in other studies 33, 38, 41, we also assessed the role of Akt signalling in this study. Our results suggested that inactivation of Akt by BBR combining with MET was involved in the reduced expressions of SP1 and DNMT1. This was in line with other reports 57, 58, 59 highlighting the key role of this kinase signalling in this process. Of note, the findings obtained from BBR and MET combination treatment suggested at least an additive effect existed in our system, the statistical significance specifically for the true synergy and the clinical significance of this combination therapy need to be determined. Nevertheless, all the results might provide some solid information for guiding the combinational treatment using natural anticancer compounds and other agents in enhancing efficiency for lung cancer treatment.

More importantly, our *in vivo* data were consistent with the findings from that *in vitro,* confirming the enhanced effect of BBR in the presence of MET on inhibition of lung cancer 8, 60, 61, and regulation of SP1, PDPK1 and DNMT1 expressions. The doses of BBR or/and MET used were based on other studies 8, 35, 42. Given the fact that the information of actual doses of these in animal blood remained to be determined, we think that further experiments are required to obtain this. In addition, more studies are also needed to further determine the role of DNMT1 in this process using cells stable transfected with shRNAs or/and exogenously DNMT1 expression vectors in nude mice model. In addition, whether BBR has potential in prolonging the survival and inhibiting metastasis in lung xenografted tumours required to be elucidated.

Collectively, our results show that BBR inhibits growth of NSCLC cells through inhibition of SP1 and PDPK1; subsequently, this results in the reduction in DNMT1 gene expression. The reciprocal interplay of PDPK1 and SP1 contributes to the inhibition of DNMT1 gene expression in response to the effects of BBR. In addition, there is a potential synergy of BBR and MET (Fig. 6F). This study reveals a novel mechanism in response to BBR in combination with MET and suggests a new strategy for NSCLC‐associated adjuvant therapy.

## Author contributions

S.S.H. is fully responsible for the study designing, experiment adjustment, drafting and finalizing the manuscript. F.Z .performed most of the experiments involved. J.J.W. carried out part of transfection assays and some protein measurement by Western blot and statistical analysis. Q.T. conducted part of densitometry, statistical analysis and participated in coordination manuscript. Q.X. executed part of the MTT assays, some overexpression experiments and statistical analysis. W.Y.W. coordinated and provided important suggestions including some reagents, and critical reading the manuscript. All authors read and approved the final manuscript.

## Conflicts of interest

The authors have declared that no competing interests exist.
